# Supplementary figures and images for: The Viral Polymerase Inhibitor 7-Deaza-2’-C-Methyladenosine Is a Potent Inhibitor of In Vitro Zika Virus Replication and Delays Disease Progression in a Robust Mouse Infection Model
Source: PLoS Negl Trop Dis. 2016 May 10;10(5):e0004695. doi: 10.1371/journal.pntd.0004695 (PMC4862633; doi:10.1371/journal.pntd.0004695)

# Supplementary Figure 1

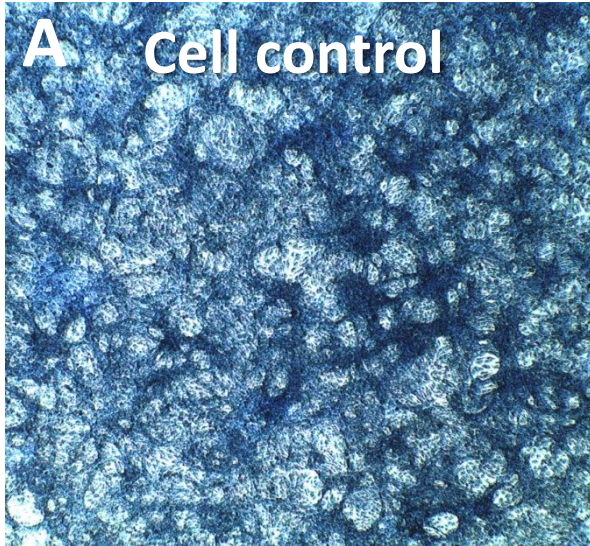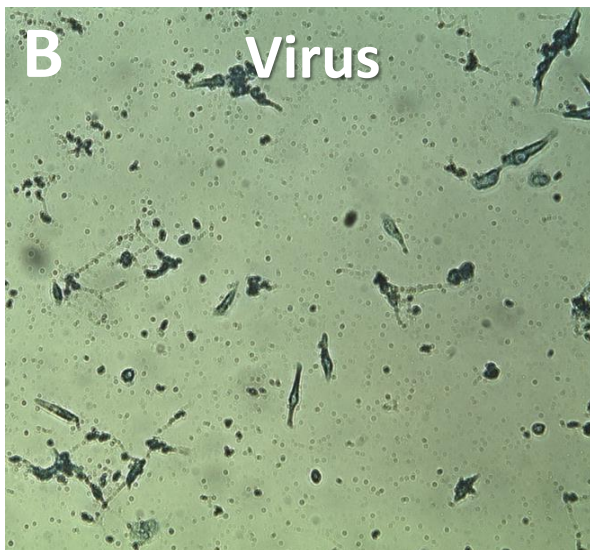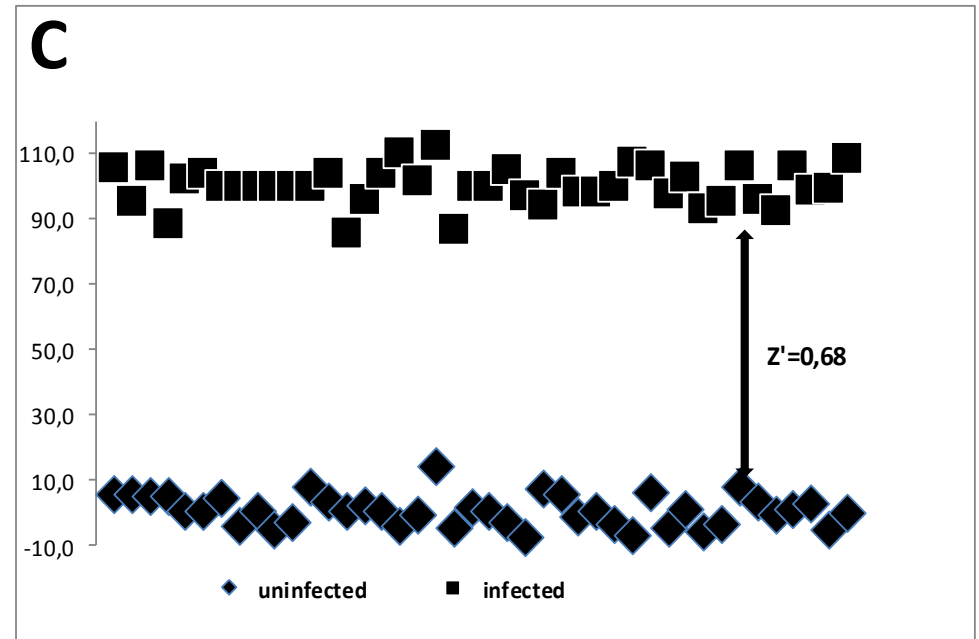

Supplement: S1 Fig — Vero cells infected with ZIKV MR766 causes full CPE (B) at day 5 pi; uninfected cells (A). Z’ factor (0.68) was calculated for 64 samples (in 8 independent experiments; C) determined by the MTS readout method using the formula: 1-[3×(SDCC+SDVC)/(ODCC-ODVC)]; VC, virus control; CC, cell control. (PDF) [file pntd.0004695.s001.pdf]

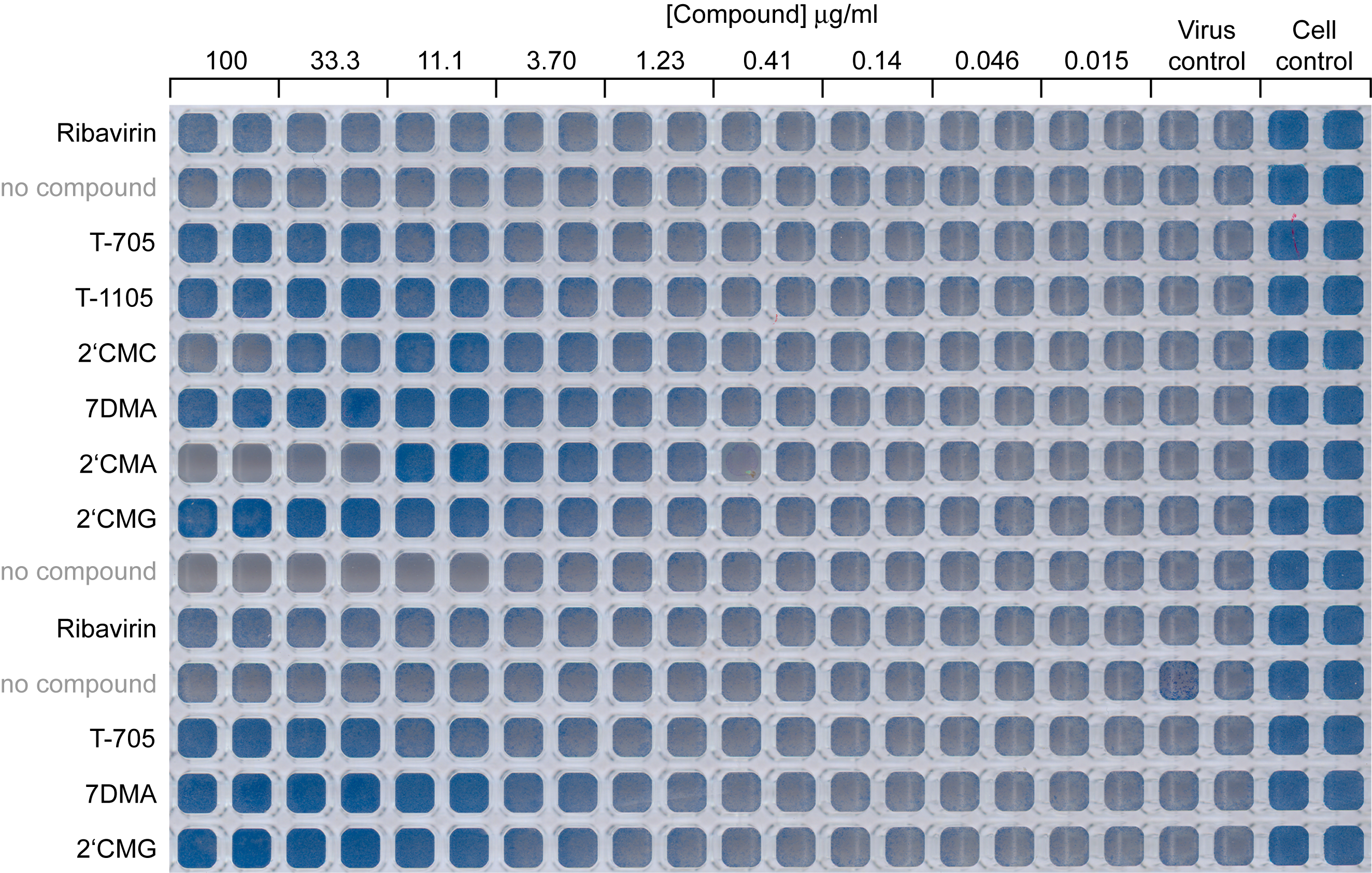

Supplement: S2 Fig — Vero E6 cells were infected with ZIKV MR766 in the presence of (3-fold serial dilutions of) a panel of compounds (2’-C-methyladenosine [2’CMA], 2’-C-methylguanosine [2’CMG], 2’-C-methylcytidine [2’CMC], 7-deaza-2’-C-methyladenosine [7DMA], ribavirin, favipiravir [T-705] and T-1105). Cells were fixed and stained using a 1% methylene blue solution at day 7 pi. (TIF) [file pntd.0004695.s002.tif]

# Supplementary Figure 4A-4D

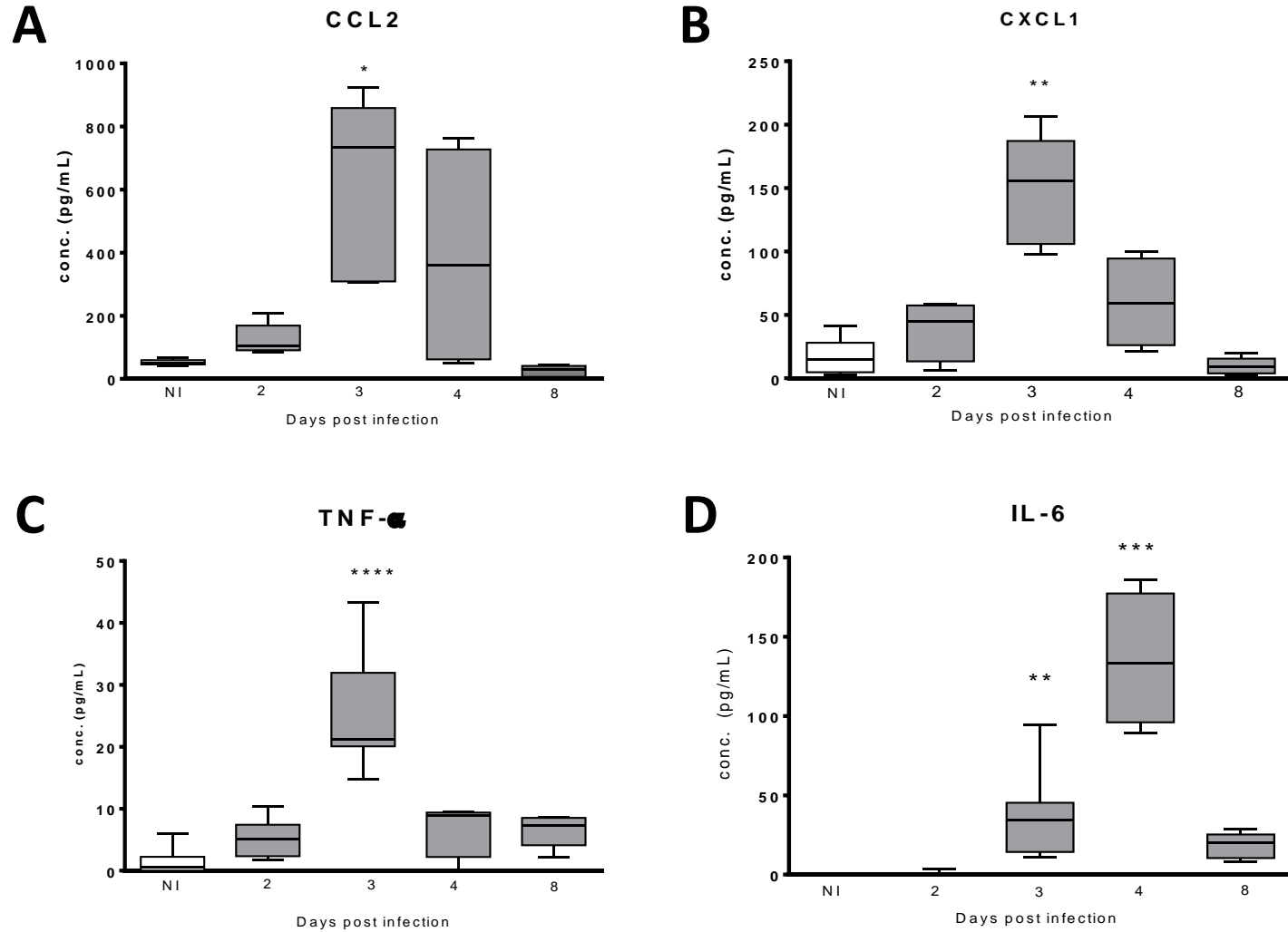

# Supplementary Figure 4E-4G

**E**

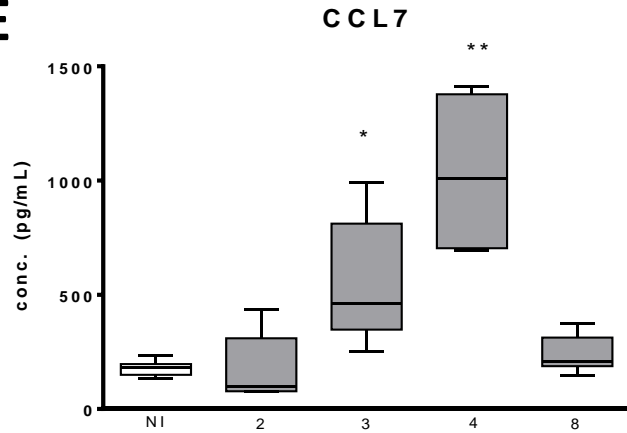

**F**

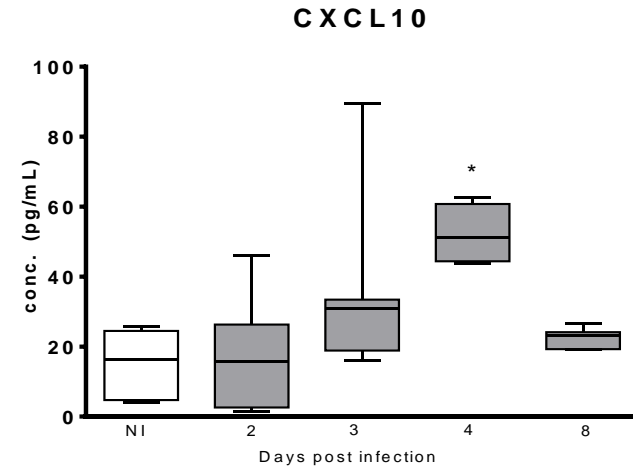

**G**

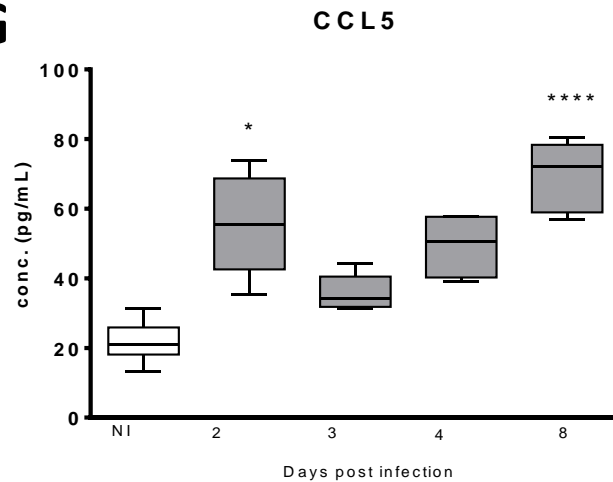

Supplement: S4 Fig — Induction of cytokines and chemokines was detected in 20 μL of serum using the ProcartaPlex Multiplex Immunoassay Panel with Mouse Th1/Th2 & Chemokine Panel 20-Plex (e-Bioscience). Statistical analysis was performed using a one-way ANOVA. *, p<0.05. (PDF) [file pntd.0004695.s004.pdf]
